# Lessons learned from the twinning TwinSubDyn collaboration

**DOI:** 10.12688/openreseurope.23059.3

**Published:** 2026-05-26

**Authors:** Snežana Maletić, Jelena Beljin, Bruno Glaser, Arthur Gross, Thilo Hofmann, Thorsten Hüffer, Heike Knicker, Marijana Kragulj Isakovski, Srđan Rončević, Lutz Weihermüller, Viktoriia Lovynska, Roland Bol

**Affiliations:** 1 University of Novi Sad Faculty of Sciences, Novi Sad, 21000, Serbia; 2 Martin Luther University Halle-Wittenberg, Institute of Agricultural and Nutritional Sciences, Soil Biogeochemistry, Halle, Germany; 3 University of Vienna, Centre for Microbiology and Environmental Systems Science, Department for Environmental Geosciences, Vienna, Austria; 4 Instituto de la Grasa, Consejo Superior de Investigaciones Científicas IG-CSIC, Seville, Spain; 5 Institute of Bio- and Geosciences, IBG-3 Agrosphere, Forschungszentrum Jülich GmbH, Julich, Germany

**Keywords:** capacity building, organic soil amendment, contaminant transport, carbon and nutrient dynamics, compost, digestate, manure, hydrochar, biochar.

## Abstract

Widening European countries face persistent limitations in scientific infrastructure, international scientific integration, and interdisciplinary training. The Horizon Europe Twinning project “Twinning excellence on organic soil amendments effect on nutrient and contaminant dynamics in the subsurface” - TwinSubDyn addressed these gaps by strengthening conceptual, analytical, and interpretive capacity at the University of Novi Sad Faculty of Sciences (UNSPMF). This was achieved through collaboration with four leading Universities and research institutes with EU: University of Vienna (UNIVIE), Forschungszentrum Jülich (FZJ), Martin Luther University Halle–Wittenberg (MLU), and Spanish National Research Council (CSIC). Focusing on organic soil amendments (OSA) and their effects on soil organic matter (SOM), nutrient dynamics, hydrology, and contaminant fate, the project operated in field-relevant conditions where OSA effects are highly context-dependent and challenging to interpret. TwinSubDyn applied integrated and concept-driven research, combining mobility of scientists, expert visits, collaborative research, trainings, workshops, and a summer school. Although the research component was modest, it provided essential focal points for training, joint interpretation, and mutual learning. Collaborative activities produced multi-scale insights into OSA aging, SOM transformations, nutrient mobility, and sorption processes. Mobility of scientists and expert exchanges substantially strengthened UNSPMF’s analytical confidence and interdisciplinary reasoning, while the partners gained comparative insights into Western Balkan scientific capacity, research organization, and region-specific environmental conditions. The project fostered new research collaborations, enhanced institutional preparedness, and broadened engagement with policymakers, practitioners, and the public. TwinSubDyn demonstrates that twinning efforts achieve greatest impact when conceptual training and applied research are tightly integrated, offering a transferable model for future scientific development initiatives.

## 1. Introduction

Widening countries are European Union (EU) Member States and associated countries that historically show lower research and innovation performance and are therefore targeted for capacity-building measures under Horizon Europe. Research capacity across the European Research Area remains unevenly distributed, with institutions in Widening countries often constrained by limited access to advanced scientific infrastructure and networks, high-level training, and political restrictions. To address these disparities, the EU did establish the Twinning scheme ( https://ec.europa.eu/info/funding-tenders/opportunities/portal/screen/opportunities/topic-details/horizon-widera-2021-access-02-01), which promotes structured collaboration between emerging institutions and internationally leading research groups. Twinning projects aim not only to enhance scientific excellence but also to foster sustainable institutional development, mutual learning, and long-term integration of Widening institutions into European research networks.

The twinning project “Twinning excellence on organic soil amendments effect on nutrient and contaminant dynamics in the subsurface - TwinSubDyn” was developed in 2021 against this framework. Thereby responding to a growing scientific and policy-relevant corporation need across both the EU and the Western Balkans. Organic soil amendments (OSA), which include compost, digestate, manure, hydrochar, and biochar, are in the EU and globally increasingly promoted for soil restoration, nutrient recycling, carbon sequestration, and climate resilience. ^1, 2^ Their usage aligns with key EU policy frameworks such as the EU Soil Strategy for 2030 (EU, 2030), the Circular Economy Action Plan, ^4^ the Farm-to-Fork Strategy, ^5^ and the Zero Pollution Ambition (EC, 2021). Yet, the use of OSAs also induce complex chemical, physical, and biological subsurface changes that influence soil organic matter (SOM) composition, dissolved organic carbon (DOC), nutrient cycling, colloid formation, microbial processes, and contaminant mobility. ^7– 14^ Understanding these interconnected effects under realistic field conditions is scientifically challenging, especially in heterogeneous or contamination-affected soils. ^15– 18^

Simultaneously, Serbia and other Western Balkan countries face differential regulatory, infrastructural, and knowledge limitations, that hinder safe, effective and uniform consistent OSA deployment. The region lacks overall harmonized product standards, advanced analytical facilities, and long-term field datasets comparable to those in Western Europe. Although, UNSPMF conducted active research in soil chemistry and OSA applications prior to the project, further enhanced conceptual, analytical, and modeling capacity was needed to fully address the environmental and regulatory complexities associated with OSA use.

TwinSubDyn was therefore designed to strengthen UNSPMF’s scientific excellence by integrating complementary expertise from four leading EU institutions: University of Vienna (UNIVIE), Forschungszentrum Jülich GmbH (FZJ), Martin Luther University Halle–Wittenberg (MLU), and Spanish National Research Council (CSIC). The project adopted a concept-driven twinning model that emphasized analytical reasoning, interpretation skills, hydrological and biogeochemical understanding, and interdisciplinary cross-partner collaborations and research staff exchanges. Given structural and infrastructural constraints in Widening contexts, this approach was preferred over laboratory replication or equipment-intensive transfer, which would not have been sustainable after the end of the project. This experience suggests that concept-driven twinning models, focused on analytical reasoning and mutual learning rather than equipment transfer, can represent an effective and sustainable approach for future Twinning and other Widening projects.

By combining research mobility, expert visits, collaborative experiments, modeling exercises, workshops, and summer school, the project sought to create a cumulative learning environment in which conceptual depth and interpretive capacity could develop across the entire UNSPMF team. In turn, EU partners deepened their understanding of Western Balkan soils, local amendment materials, and region-specific environmental pressures, enabling mutual scientific learning rather than one-directional capacity transfer.

This paper aims to systematically analyze the TwinSubDyn twinning project as a case study of research capacity building in a Widening country. Specifically, it (i) evaluates how key twinning activities (mobility, collaborative research, expert training, and stakeholder engagement) contributed to strengthening research capacity at UNSPMF, (ii) synthesizes the main scientific and institutional outcomes of the project, and (iii) reflects on lessons learned and future directions for designing effective twinning-based capacity building strategies within the European Research Area. This paper synthesizes the final scientific, institutional, and collaborative learning that emerged from TwinSubDyn. Rather than reflecting on administrative and research-based outputs, we focus on how the project shaped research practices, scientific interpretation, and institutional evolution and collaboration. The following sections describe the methodological framework of the project, the results of mobility and research activities, and the cross-cutting lessons learned, that can inform future widening actions and twinning collaborations across Europe.

## 2. Methodological approach

### 2.1 Overall concept

TwinSubDyn applied a multi-layered methodological design that combined mobility, conceptual exchange, expert visits, collaborative research, and structured training. Instead of relying on isolated interventions, the project built an integrated capacity-building framework, in which knowledge acquisition occurred through repeated, complementary engagements. This design also facilitated a shared understanding among partners by newly exposing them to Western Balkan soil systems, environmental pressures, and regulatory contexts.

Although the research component of TwinSubDyn was necessarily limited in scope due to time and budget constraints, it played a crucial role in structuring the capacity-building process. The joint experiments, lysimeter studies, modeling exercises, and shared analytical activities provided concrete focal points around which mobilities, expert visits, and training were organized. Without such an applied research core, the twinning outcomes would likely have been significantly weaker, as seen in initiatives such as COST actions, where mobility occurs without actual shared experimental work. In TwinSubDyn, even a modest research component ensured that learning remained anchored in real datasets, site-specific challenges, and joint scientific interpretation.

### 2.2 Conceptual framework for mutual learning

The project’s approach to capacity building was based on three guiding principles:

(i) Exposure to advanced scientific expertise across several European laboratories
(ii) Iterative knowledge accumulation through repeated exchanges, and
(iii) Mutual learning generated through collaboration on shared research questions.

**Figure 1.**
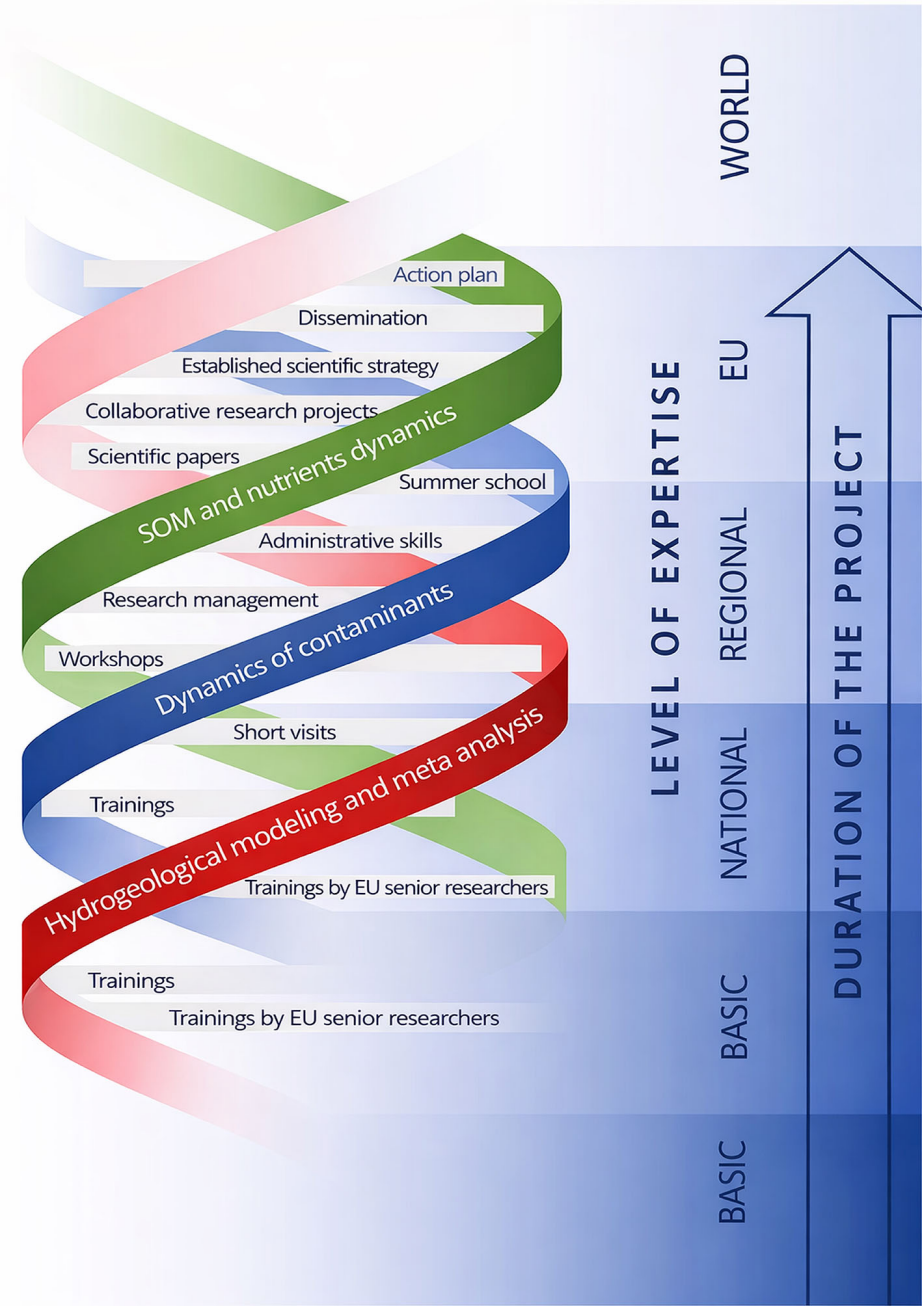
TwinSubDyn conceptual approach.

**Figure 2.**
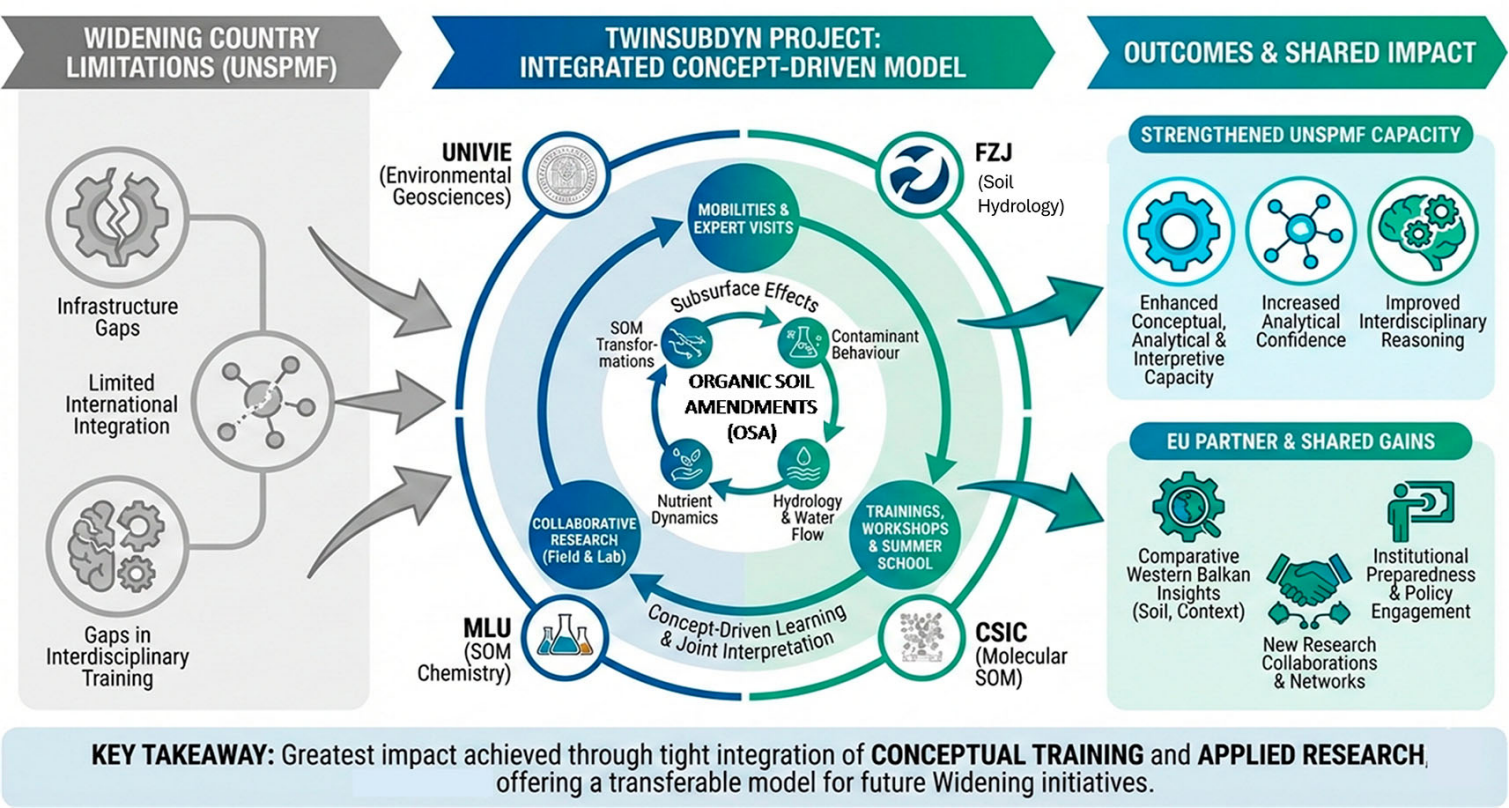
Visual summary of the TwinSubDyn learning and capacity-building model.

Figures 1 and 2 illustrate the adopted conceptual learning model, showing how mobilities, expert visits, collaborative research, and workshops interact to create cumulative learning effects. In applying this framework, the consortium learned that an open, concept-driven approach created multiple entry points for learning across all career stages, from MSc students and PhD candidates to postdoctoral researchers and senior academics. Unlike models centered on narrow technical transfer, this structure enabled a broad distribution of knowledge acquisition and interpretation skills within UNSPMF. It also facilitated true mutual learning across partners, as researchers with different backgrounds engaged with shared data and conceptual questions. Moreover, the openness of the approach allowed interactions not only within the consortium but also with practitioners, stakeholders, and the general public, particularly through workshops and field demonstrations. These experiences show that knowledge gained through TwinSubDyn extended beyond laboratory or modeling tasks and supported wider scientific, institutional, and societal learning.

The four partner institutions contributed distinct scientific and methodological capacities, each corresponding to specific learning needs at UNSPMF. Their expertise and contributions are summarized in Table 1.

**Table 1.** Overview of partner expertise and main learning contributions for UNSPMF.

Partner institution	Core expertise	Main contribution to UNSPMF learning
**UNIVIE (Austria)**	Environmental geosciences, organic contaminants, colloids, nanoparticles, hydrological modeling.	Exposure to contaminant behavior, nanoparticle and colloid transport concepts, and hydrological modeling logic.
**FZJ (Germany)**	Soil hydrology, isotope applications, nutrient & carbon cycling, modeling unsaturated flow.	Understanding water flow–solute transport interactions, lysimeter interpretation, and isotope-based tracing principles.
**MLU(Germany)**	SOM chemistry, long-term OSA effects, fractionation methods, isotope-based SOM turnover.	Conceptual insight into SOM stabilization, amendment aging, and interpretation of long-term field data.
**CSIC (Spain)**	Molecular SOM characterization (solid-state NMR, pyrolysis-GC/MS), organic geochemistry.	Exposure to molecular-level SOM analysis, amendment-induced compositional changes, and organic matter recalcitrance.

### 2.3 Modes of knowledge acquisition

TwinSubDyn implemented a variety of multiple learning mechanisms addressing different types of knowledge and levels of expertise.

**Short-Term Mobilities -** Short visits (up to one month) enabled UNSPMF senior researchers to observe partner workflows, conceptual approaches, and analytical strategies. These stays were primarily conceptual, offering high-level exposure to advanced research environments rather than hands-on training.

**Long-Term Mobilities for Early-Career Researchers -** Extended visits (up to six months) allowed PhD and early-career researchers to engage more deeply with specific analytical or modeling tasks, participate in active research, and develop confidence in experimental interpretation and scientific reasoning.

**Expert Visits to UNSPMF -** Expert visits delivered targeted conceptual training through lectures, demonstrations, hydrological modeling exercises, and field-based demonstrations. These activities broadened understanding of analytical and modeling principles without duplicating laboratory infrastructure.

**Collaborative Research -** Joint analytical work, shared experiments, and combined modeling efforts served as an applied learning process. This mode enabled UNSPMF researchers to generate and interpret data collaboratively with EU partners, reinforcing conceptual understanding and analytical reasoning.

**Workshops and Summer School -** Workshops provided structured exchange with experts, policymakers, and practitioners, while the summer school created an intensive interdisciplinary environment for scientific, regulatory, and methodological training.

These different learning mechanisms and their functions are summarized in Table 2.

**Table 2.** Components of the TwinSubDyn learning model.

Learning mechanism	Purpose	Type of learning	Primary beneficiaries
**Short-term mobilities**	Exposure to partner workflows and conceptual frameworks	Conceptual familiarization	Senior researchers
**Long-term mobilities**	Deep engagement with research and modeling processes	Applied conceptual & partial practical	PhD/ECRs
**Expert visits**	Targeted conceptual training at UNSPMF	Conceptual/systemic	Entire UNSPMF team
**Collaborative research**	Joint data generation and interpretation	Applied, analytical, integrative	All partners
**Workshops & Summer School**	Interdisciplinary exchange & stakeholder engagement	Interdisciplinary, communication, policy	UNSPMF, partners, stakeholders

### 2.4 Data sources

We synthesized the information from mobility reports, research datasets, workshop materials, expert-visit reflections, summer school evaluations, and cross-partner discussions. The meta-synthesis integrated multiple project outputs (D1.2, D1.3, D2.1, D3.1, D3.2, D1.5, and the Summer School report) to identify common findings and differences across activities. The analysis included screening key outcomes, grouping findings into core domains, and interpreting links between activities, outcomes, and lessons learned. Material are available at https://twinsubdyn.pmf.uns.ac.rs/; https://knowledge-hub.pmf.uns.ac.rs/; https://zenodo.org/search?q=twinsubdyn&l=list&p=1&s=10&sort=bestmatch; https://zenodo.org/me/uploads?q=&f=shared_with_me%3Afalse&l=list&p=1&s=10&sort=newest.

## 3. Results and analysis

### 3.1 Mobility as a catalyst for learning

**3.1.1 Mobility approach**

**Figure 3.**
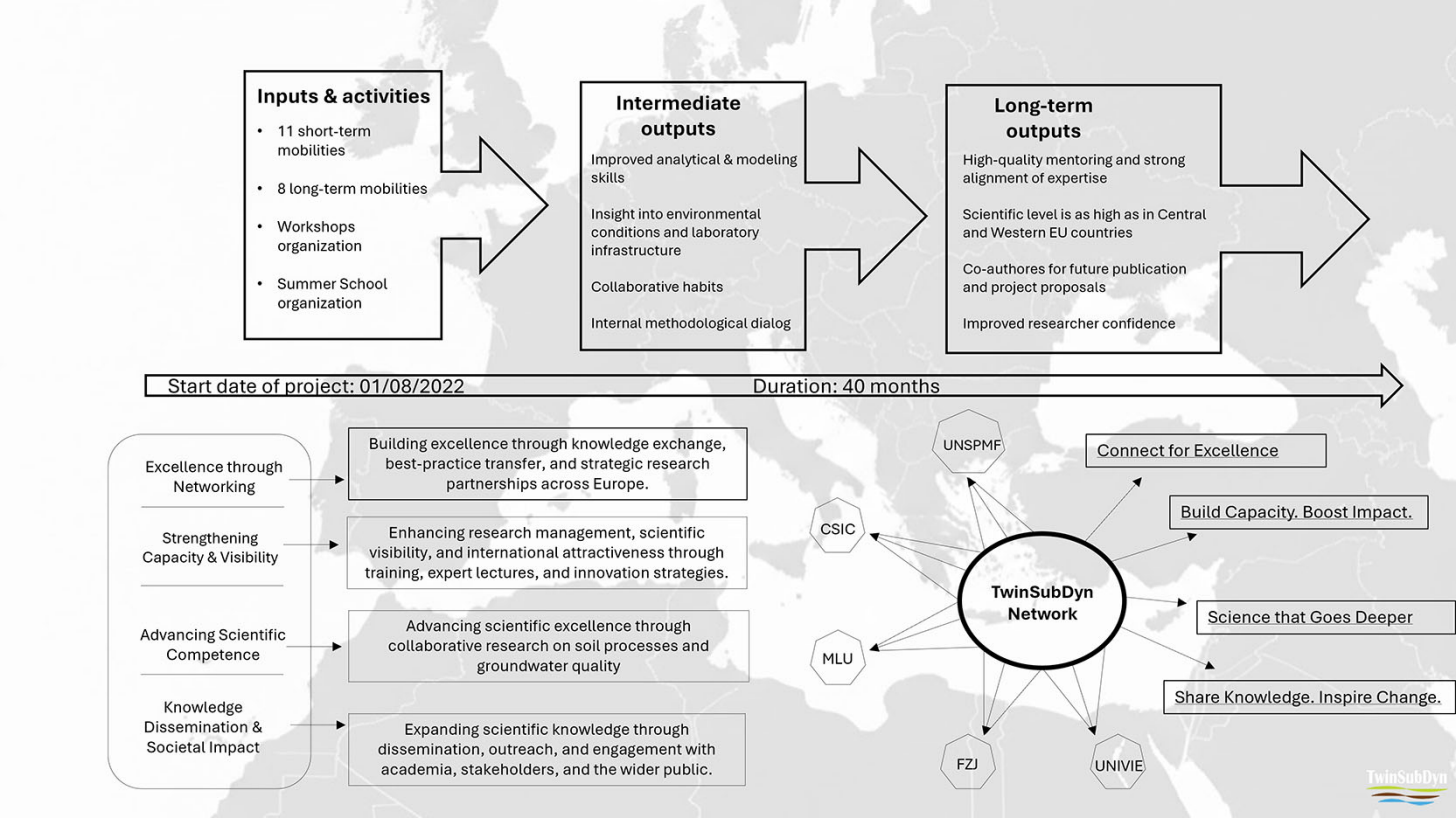
TwinSubDyn mobility scheme and resulting learning and capacity-building outcomes.

Mobility formed the backbone of TwinSubDyn’s capacity-building strategy, providing structured opportunities for UNSPMF researchers to engage directly with partner institutions and their scientific environments. Across the project, a total of 11 short-term mobilities and eight long-term mobilities were completed, involving senior researchers, postdoctoral fellows, and PhD candidates ( Figure 3). These exchanges enabled conceptual learning, exposure to advanced analytical and modeling workflows, and deeper understanding of issues regarding amendment–soil interactions across different European research contexts.

The mobilities were not intended to replicate laboratory infrastructure or build technical capacity through equipment-intensive training. Instead, they served as immersive observational and interpretive experiences that strengthened analytical thinking, scientific reasoning, and collaborative habits of work. For UNSPMF researchers, this represented the first sustained exposure to high-level research environments in several domains: solid-state NMR and molecular SOM characterization (CSIC), SOM fractionation and isotopic approaches (MLU), contaminant dynamics and nanoparticle behavior (UNIVIE), hydrological modeling and setup and interpretation of lysimeter experiments (FZJ). At the same time, mobilities provided EU partners with new valuable insights into Western Balkan soils, local OSA materials, and region-specific environmental pressures. These differences in soil contamination history, hydrological patterns, and amendment characteristics offered comparative perspectives seldom encountered in Central and Western European laboratories.

**Short-Term Mobilities** (up to one month) allowed senior UNSPMF staff to align conceptual frameworks with partner institutions, observe data interpretation workflows, and participate in targeted discussions on experimental design and modeling logic. These stays played an important role in building shared understanding and facilitating future collaboration.

**Long-Term Mobilities** (up to six months) involved mainly PhD students and early-career researchers. They provided meaningful participation in sample preparation, instrument workflows (where feasible), data processing, and joint interpretation sessions. These extended stays strengthened scientific independence, improved communication within international teams, and contributed directly to integrated data interpretation across the consortium.

**Expert visits** brought partner expertise directly to UNSPMF offering targeted training in: microplastic sample preparation, LC–MS/MS workflows, solid-state NMR principles, modeling, colloid and nanoparticle transport, lysimeter experiments, and field sampling. Because these activities occurred on-site, they were directly embedded within UNSPMF’s research reality, enabling researchers who could not travel to engage in training and strengthening the internal methodological dialogue within the team.

**3.1.2 Strengths and challenges**

Mobilities were overall successful, generating continuity between training and research and strengthening interpersonal and institutional links. The major strength was the precise alignment between partner expertise and specific UNSPMF needs. Challenges arose mainly from structural and logistical constraints: administrative procedures, scheduling issues during busy laboratory periods, steep learning curves for early-career researchers entering specialized analytical environments, and limited access to instruments at specific times. Despite these constraints, mobilities contributed substantially to building UNSPMF’s analytical confidence, interpretive capacity, and collaborative readiness. Comparative partner perspectives on mobility activities are given in Table 3.

**Table 3.** Comparative perspectives on mobility activities.

Question	UNSPMF	UNIVIE	FZJ	MLU	CSIC
Key benefit of mobility	Improved analytical/modeling skills and researcher confidence.	Exposure to diverse soil systems and OSA expanded our dataset and stimulated comparative thinking beyond Central European conditions.	Connecting to young researchers and research interest outside existing networks.	Insight into environmental conditions and laboratory infrastructure.	Enhanced interdisciplinary collaboration with UNSPMF and among the participant partners.
Main strength observed in collaborators	High-quality mentoring and strong alignment of expertise.	UNSPMF researchers showed strong conceptual rigor and an impressive ability to integrate hydrological, chemical, and geochemical perspectives.	To learn about the specific regional problems and needs.	Scientific level is as high as in Central and Western EU countries.	Deep technical knowledge and scientific background and proactive collaboration.
Biggest challenge during exchange	Admin load, steep learning curves, scheduling constraints.	Synchronizing analytical schedules with ongoing laboratory commitments limited the time available for extended hands-on instrument use.	To use the given (short) time most effectively, especially for experiments.	Time and budget constraints.	Navigating complex instrumentation and adapting lab protocols. Scheduling constrains.

**3.1.3 Synthesis and mutual learning**

Through repeated interactions, UNSPMF researchers accumulated a deeper understanding of how advanced analytical and modeling tools are applied in leading laboratories. For partner institutions, exposure to Western Balkan soil systems offered comparative insights not typically represented in established datasets they work with. Mobilities thus created an interconnected learning process that supported joint data interpretation and strengthened scientific cohesion across the consortium. This shared experience lays the foundation for future collaborations and co-authored publications.

### 3.2 Research activities

**3.2.1 General contributions overview**

TwinSubDyn project resulted in an integrated, multi-scale dataset through coordinated contributions from all partners:

• **UNSPMF**: soil and amendment characterization, biochar/hydrochar production, pot experiments, Serbian lysimeter operation, nutrient and pesticide analysis.
• **CSIC**: molecular SOM characterization (solid-state NMR, analytical pyrolysis, isotope approaches).
• **MLU**: long-term field sampling and SOM molecular marker assessments, stable isotope analysis, high-resolution mass spectrometry.
• **FZJ**: nutrient cycling analyses, hydrological interpretation, and unsaturated zone modeling.
• **UNIVIE**: contaminant behavior, DOC and colloid dynamics, nanoparticle transport, and pollutant transport modeling support.

The collaborative research encompassed a range of research topics, several of which were new to the partners who typically address OSA-related questions from more specialized perspectives. Many of these topics would not have been addressed without the joint collaboration enabled by the twinning framework. The main topics addressed in this manner are outlined below.

**3.2.2 Baseline soil and amendment characteristics**

The various soils analyzed included sandy loam and silty clay loam soils used for lysimeter experiment, and agricultural topsoil used in controlled pot experiments at UNSPMF, and long-term field soils from the Bayreuth biochar experiment at MLU. These soils differ in texture, nutrient status, contamination background, and hydrological behavior, offering a diverse testbed for evaluating OSA effects. Used OSA displayed clear physicochemical contrasts. Biochar and co-composted biochar exhibited higher aromaticity and structural stability, while compost and hydrochar contained more labile carbon fractions and readily available nutrients. These baseline traits were essential for interpreting downstream behavior in SOM dynamics, nutrient mobility, and contaminant interactions.

**3.2.3 SOM dynamics and amendment aging**

Short-term experiments at UNSPMF demonstrated clear OSA-specific behaviors. Labile materials such as compost and hydrochar increased light SOM fractions and elevated DOC early in the experiment, while biochar-based amendments remained structurally stable within the short observation period. Long-term analysis of the Bayreuth field experiment provided a contrasting picture, revealing significant aging processes that altered amendment properties over time. Co-composted biochar exhibited increased oxygen-containing functional groups and evidence of microbial reworking, while compost-rich treatments showed deeper incorporation into microbial processed SOM pools. These findings illustrated that short-term studies cannot reliably predict long-term OSA behavior and that multi-year monitoring is essential for understanding OSA evolution. ^8, 9, 14^

**3.2.4 Nutrient mobility and hydrological effects**

Lysimeter studies highlighted the interaction between OSA chemistry and hydrological processes in governing nutrient mobility. Nutrient leaching varied substantially among treatments and was strongly shaped by site-specific hydrological conditions. Biochar-containing OSA modified water flow behavior in some treatments, although not consistently across soils or seasons. Compost and hydrochar, which released larger amounts of DOC, generated more variable nutrient leaching patterns that reflected both OSA chemistry and seasonal hydrological dynamics. Hydrological modeling conducted by FZJ clarified differences in soil water movement and provided essential context for interpreting nutrient flux patterns, even though nutrient transformations themselves were not modeled. ^19^

**3.2.5 Contaminant behavior and emerging processes**

OSA effects on contaminant behavior were similarly context dependent. Changes in DOC composition, soil surface properties, and ionic strength influenced sorption environments in ways that varied by soil type and compound. Sorption–desorption tests and column experiments with selected pesticides showed that OSA sometimes could increase, decrease, or have negligible effects on contaminant mobility. These outcomes highlighted the need for site-specific assessment rather than reliance on generalized expectations. ^7, 11– 13^ Preliminary studies of microplastics and colloids suggested emerging research directions, including interactions between OSA-derived DOC and microplastic-associated compounds, and potential amendment-driven changes in pore architecture affecting colloid mobility. ^10^

**3.2.6 Integrative value of modeling approaches**

Modeling served primarily as an interpretive tool in the project. Hydrological modeling focused on water flow and retention patterns, illustrating how amendment-induced changes in hydraulic properties influenced infiltration, evaporation, and drainage. UNSPMF’s exploratory contaminant transport modeling used sorption coefficients and retardation factors to examine how changes in DOC, sorption behavior, and pore structure might influence pesticide mobility. Although, these models were preliminary and not calibrated to field conditions, they helped clarifying mechanisms underlying experimental observations and strengthened UNSPMF’s conceptual understanding of amendment–soil interactions.

**3.2.7 Synthesis of scientific learning**

Three overarching insights emerged from the integrated research activities. First, OSA effects on SOM dynamics, nutrient mobility, and contaminant behavior were highly context-dependent, shaped by amendment chemistry, soil properties, hydrological conditions, and contamination history. Second, aging processes fundamentally altered amendment functionality, demonstrating that long-term datasets are indispensable. Third, mechanistic understanding of amendment effects required integration across molecular, laboratory, lysimeter, field, and modeling scales, confirming the value of the consortium’s multi-scale approach.

Together, these findings highlight the importance of holistic research frameworks for evaluating amendment performance across diverse environmental settings ( Table 4).

**Table 4.** Integrated summary of research results.

Component	Key findings	Interpretation
Amendment Properties	Biochar/co-composted biochar: aromatic, stable. Compost/hydrochar: labile, DOC- and nutrient-rich.	Chemistry determines short- vs long-term behavior.
Short-Term Soil Responses	Labile OSAs increased DOC and light SOM fractions; biochar remained stable.	Early effects reflect intrinsic amendment traits.
Aging Effects (Field)	Biochar showed long-term stability but structural changes; compost-rich materials showed enhanced microbial processing.	Aging alters amendment function; long-term data essential.
Nutrient Mobility	Nutrient leaching varied by soil and amendment; no consistent reduction under biochar.	Hydrology largely controls nutrient fate.
Contaminant Behavior	OSAs modified sorption and availability; effects compound- and soil-specific.	No universal pattern: case-specific assessment needed.
Emerging Processes	Preliminary signals of DOC–microplastics interactions and colloid mobility shifts.	Early-stage directions for future research.
Modeling (Hydrology & Transport)	Hydrological modeling explained water flow differences; UNSPMF transport modeling explored pesticide mobility.	Provides mechanistic context for interpreting experiments.

### 3.3 Capacity-building events and institutional learning

Workshops, the summer school, and administrative-support webinars complemented the scientific and mobility activities and played a major role in strengthening institutional capacity at UNSPMF. Scientific workshops allowed partners to exchange expertise, harmonize methodological approaches, and jointly interpret emerging results related to OSA and SOM dynamics, hydrological processes, and contaminant fate. Later workshops expanded to regulatory, market, and stakeholder dimensions, revealing persistent barriers to safe and effective OSA deployment in the Western Balkans, including gaps in product standards, weak regulatory enforcement, and limited user awareness.

The summer school provided an intensive interdisciplinary learning environment that combined conceptual lectures with practical demonstrations and case-based discussions. Feedback from participants highlighted the value of integrating OSA chemistry, hydrological interactions, contaminant behavior, and policy considerations into a single training framework. Administrative-support webinars strengthened internal UNSPMF procedures related to FAIR data (findable, accessible, interoperable, and reusable), communication, branding, technology transfer, project development, and institutional coordination.

To capture the diversity of experiences across the consortium, Table 5 presents concise partner reflections on how the workshops, summer school, and webinars contributed to their scientific and institutional learning. Together, these activities reinforced scientific, organizational, and strategic preparedness, ensuring that capacity building extended beyond scientific training to the broader institutional systems required for effective EU research participation.

**Table 5.** Partner perspectives on workshops, summer school, and webinars.

Institution	What was most valuable for your team?	What did you learn about the Western Balkan context or UNSPMF needs?
**UNSPMF**	These events helped build confidence, improve internal coordination, and strengthen our ability to engage in European research networks.	The activities confirmed that UNSPMF’s key needs lie in conceptual strengthening, interdisciplinary integration, and improved institutional support rather than direct technical replication.
**UNIVIE**	The workshops created space for joint interpretation of contaminant–DOC interactions, which provided new comparative insights across soil types.	We gained a clearer understanding of the region’s heterogeneous contamination history and the need for stronger long-term monitoring frameworks.
**FZJ**	Events helped to develop networks, which are useful for future project applications.	Participants often perform excellent research but partly lack international visibility. UNSPMF needed to be more oriented to controlled field and lysimeter experiments.
**MLU**	Enhancing teambuilding and distribution of standardized training material.	I learned that UNSPMF is surprisingly well developed and scientific level is comparable to the one in Central and Western EU countries. Therefore, it was no twinning project but a real scientific collaboration at the same scientific level.
**CSIC**	The exchange events helped to enhance networking not only among IPs but also among the early carrier scientists. It opened the door to extend our collaboration from more central Europe orientated towards Western Balkan.	We broadened our horizon by recognizing the specific challenges of environmental issues regarding contaminated soils in Serbia and the need of UNSPMF to integrate and network in European research networks.

In addition to the structured institutional perspectives captured in Table 5, it was important to document how the learning framework was experienced individually across different groups involved in TwinSubDyn. The anonymous reflections, presented in Table 6, illustrate how PhD candidates, postdoctoral researchers, senior scientists, administrative staff, external trainers, practitioners, and members of the general public perceived the learning environment and collaborative experience.

**Table 6.** Examples of learning experiences across participant groups (anonymous reflections).

Participant group	What was learned (Anonymous reflection; max. 2 lines)
PhD students	“My first exposure to European laboratories showed me how my research connects to broader soil processes.” “Working with multiple partners helped me understand data interpretation and improved my confidence enormously.”
Postdoctoral researcher – ERC scientist	“Twinning pushed me to think across disciplines, not just within analytical chemistry.” “The project enabled genuine co-creation rather than one-directional knowledge transfer.” “Working with partners helped us move beyond purely chemical interpretations and understand amendment impacts within hydrological and biogeochemical frameworks.”
Senior UNSPMF researcher	“We gained a clearer sense of how international consortia function, which will help us coordinate future EU projects more effectively.” “Repeated exchanges showed us how small conceptual shifts can significantly improve experimental design and data interpretation.”
Administrative staff	“We improved our understanding of EU procedures, reporting, FAIR data and internal coordination.” “The exposure to different analytical philosophies across partners broadened our scientific ‘toolbox’ far beyond laboratory methods we previously relied on.”
EU partner researcher	“UNSPMF is an excellent partner for research, as the labs are well equipped and personal is well trained. Additionally, the openness for integrated perspectives and cooperation helped to make the project a success.” “The collaboration broadened our perspective on how soil and amendment behavior varies across contrasting European contexts.” “Working with UNSPMF highlighted the scientific potential of Western Balkan teams and the value of more inclusive research networks.” “UNSPMF is surprisingly well developed and scientific level is comparable to the one in Central and Western EU countries. Therefore, it was no twinning project but a real scientific collaboration at the same scientific level.” “We gained deeper insights into the problematic of aquifer contamination and the importance of being integrated into a European Research Network.”
Industrial and policy/stakeholder representative	“The workshops clarified the scientific basis of OSA use and risks in practice.” “The project clarified which soil risks require stricter oversight, and which practices can be safely promoted within national sustainability strategies.”

## 4. Mutual learning, institutional change, and future research priorities

### 4.1 General outcome

TwinSubDyn demonstrated that targeted twinning partnerships can generate substantial and lasting institutional change when mobility, expert exchange, collaborative research, and stakeholder engagement are strategically integrated. Instead of focusing on the replication of specialized laboratory techniques, which would have been neither feasible nor sustainable, the project prioritized conceptual, analytical, and interpretive capacity building. This approach proved central to strengthening UNSPMF’s ability to work with complex multi-scale datasets and to interpret amendment–soil interactions in a scientifically rigorous and interdisciplinary manner.

### 4.2 Changes in scientific practice at UNSPMF

Across the consortium’s activities, it is apparent that UNSPMF researchers developed a more multidimensional understanding of OSA, moving from predominantly chemical interpretations to integrated perspectives that incorporate molecular transformations, hydrological behavior, biogeochemical cycling, and policy implications. Long-term mobilities, particularly for PhD candidates and early-career researchers, were instrumental in building scientific maturity, strengthening analytical reasoning, and improving confidence in research interpretation. These skills remain embedded within the institution and represent one of the project’s most durable outcomes.

### 4.3 Reciprocal learning within the consortium

A key dimension of TwinSubDyn was the insight partners gained into the scientific landscape of the Western Balkans. The project demonstrated that institutions in the region, despite working with limited infrastructure, fragmented monitoring systems, and complex environmental pressures, possess strong scientific potential and the capacity to engage as credible, reliable partners in European collaborations. Exposure to UNSPMF’s research environment, commitment, and scientific ambition gave partner institutions a clearer understanding of the region’s strengths, as well as of the structural barriers that continue to constrain research development. This awareness strengthened mutual respect within the consortium and highlighted the importance of sustained support for integrating Western Balkan science more fully into the European Research Area.

### 4.4 Importance of long-term processes in OSA research

TwinSubDyn also revealed the central importance of long-term processes. Analyses of multi-year field experiments demonstrated that OSA aging, SOM transformation, and nutrient and contaminant dynamics evolve substantially over time, shaping OSA effectiveness and environmental risk. These insights underscore, that short-term or isolated studies cannot reliably predict long-term outcomes and highlight the need for sustained multi-year monitoring frameworks. Exposure to hydrological and contaminant transport modeling further strengthened UNSPMF’s ability to interpret experimental data and to generalize findings across soil types.

### 4.5 Institutional strengthening beyond scientific skills

The project’s workshops, summer school, and administrative-support webinars broadened the collaboration beyond scientific training. These events enhanced UNSPMF’s internal coordination, improved understanding of FAIR data and open-science practices, strengthened proposal-writing and communication skills, and clarified regulatory and market barriers to OSA deployment in the Western Balkans. The combination of scientific and institutional strengthening increased UNSPMF’s readiness for future EU research participation.

### 4.6 Future research priorities

Continued and future collaborations will focus on further generating the long-term evidence needed to support safe and effective use of OSA across Europe, including the Western Balkans. This includes multi-year monitoring of OSA aging, coordinated cross-site trials to compare performance under contrasting environmental conditions, and modeling tools that can guide regulatory and land-use decisions. Strengthening research capacity within the Western Balkans, particularly in monitoring, modeling, and interdisciplinary assessment, remains a critical component for ensuring that the region is fully represented in European soil and climate initiatives.

### 4.7 Limitations of the study

While this study provides integrated insights into the outcomes of the TwinSubDyn project, several considerations should be taken into account when interpreting the findings. First, the analysis is primarily based on project deliverables and internal reports, which may place greater emphasis on implemented activities, while some challenges or less effective elements may be less visible. Second, the evidence is largely qualitative and context-specific, reflecting the particular institutional setting of UNSPMF and its partner network, which may limit direct generalization to other Widening institutions. Third, although the study integrates multiple sources of information, it does not include independent external evaluation or quantitative performance indicators of capacity improvement. Despite these considerations, the consistency of findings across multiple project components and data sources provides a solid basis for the conclusions and offers valuable insights for the design of future twinning initiatives.

## 5. Conclusions

TwinSubDyn clearly demonstrated that:

(i) Research capacity in Widening countries can be strengthened most effectively through integrated, concept-driven collaboration rather than through singular narrow technical transfer. By combining mobility, expert exchange, collaborative research, and stakeholder engagement, the project reshaped how UNSPMF researchers conceptualized soil processes, interpret amendment–soil interactions, and situate their work within broader European scientific and regulatory contexts.
(ii) The project opened international pathways for researchers at different career stages. Many UNSPMF PhD candidates, postdoctoral researchers - early-career academics, had never previously travelled to or conducted research within leading EU laboratories. Through TwinSubDyn, they worked abroad, built confidence, and established professional networks that will last long beyond the project’s completion. This lowered the psychological and practical threshold for future international mobility and expanded the sense of what is possible within their scientific careers. Simultaneously, more senior researchers and EU partners likewise benefited from intensive interdisciplinary interaction that broadened collaborative thinking and produced new shared research directions.
(iii) The twinning model also generated momentum for further joint research activities and several new EU-based project proposals. Trust built through sustained collaboration has strengthened the likelihood that Serbia and other Western Balkan partners can be fully integrated into future European research initiatives, not only on OSA but across broader environmental and sustainability topics.
(iv) The project also initiated outreach to new partners within and beyond the EU, and deepened interactions with industry, government agencies, NGOs, and practitioners working on soil amendment issues, thereby widening the project’s impact into policy and practice spheres.
(v) The reciprocal nature of the partnership ensured that EU institutions also gained new comparative valuable insights and knowledge, particularly regarding the environmental conditions, soil types, and amendment materials characteristic of the Western Balkans. This reciprocal exchange created a balanced partnership and established a strong foundation for continued research collaboration. Ultimately, the success of TwinSubDyn illustrates that twinning can serve as a catalyst for mutual scientific growth, institutional evolution, and durable integration into the European Research Area.

## Ethics and consent

Ethical approval was not required for this study. Case study represents overall experience regarding the implementation of Twinning project.

## Data Availability

This article is a qualitative learning and case-study report focusing on capacity building, mobility, and institutional development within the Horizon Europe Twinning project *TwinSubDyn.* It does not report new quantitative experimental results, statistical analyses, or reproducible datasets. The study synthesizes insights derived from project documentation, mobility reports, workshop materials, expert visit reports, and joint reflections generated within the TwinSubDyn consortium. These materials are not standalone research datasets but are available through publicly accessible project repositories and deliverables. Public project outputs, reports, and training materials are available at:
−
https://twinsubdyn.pmf.uns.ac.rs/
−
https://knowledge-hub.pmf.uns.ac.rs/
−
https://zenodo.org/search?q=twinsubdyn
