# Supplementary material for: Lessons learned from the twinning TwinSubDyn collaboration
Source: Open Res Eur. 2026 May 26;6:61. Originally published 2026 Feb 27. [Version 3] doi: 10.12688/openreseurope.23059.3 (PMC13150439; doi:10.12688/openreseurope.23059.3)
Supplement: Peer review history

# Reviewer report: Reviewer response for version 2

**DOI:** 10.21956/openreseurope.25676.r73276

**Published:** 2026-05-06

**Authors:** Zijian Li

**Affiliations:** 1 Sun Yat-Sen University, Shenzhen, China

I accept this manuscript and approve of its indexing.

Is the case presented with sufficient detail to be useful for teaching or other practitioners?

Yes

Is the work clearly and accurately presented and does it cite the current literature?

Yes

If applicable, is the statistical analysis and its interpretation appropriate?

Yes

Are all the source data underlying the results available to ensure full reproducibility?

Yes

Are the conclusions drawn adequately supported by the results?

Yes

Is the background of the case’s history and progression described in sufficient detail?

Yes

Reviewer Expertise:

Pesticide

I confirm that I have read this submission and believe that I have an appropriate level of expertise to confirm that it is of an acceptable scientific standard.

# Reviewer report: Reviewer response for version 2

**DOI:** 10.21956/openreseurope.25676.r73340

**Published:** 2026-05-06

**Authors:** Hai Lin Zhang

**Affiliations:** 1 China Agricultural University, Beijing, Beijing, China

 This paper is a case study with great practical significance and policy-guiding value. This article systematically reviews the experience of the TwinSubDyn project in Serbia, the Western Balkans, under the framework of "Horizon Europe". Breaking away from the traditional mindset of "one-way technical assistance" or "hardware transfer", the authors summarize a paired cooperation model centered on "Concept-driven" and "Mutual learning", which has a strong demonstrative effect on the scientific research development of the European Research Area (ERA), especially for widening countries. The authors have well incorporated the comments from the previous reviewers. However, there are still some minor issues with language and formatting, and I recommend acceptance after minor revisions.

 1. Please carefully check the contents in Table 3 and Table 5 to confirm their accuracy.

 2. At the end of the first paragraph of Section 2.1: "...environmental pressures, ures, and regulatory contexts." It is suggested to delete "ures,".

 3. Given that multiple dimensions of limitations (such as data type, subjectivity, etc.) are discussed in the paper, it is suggested to change the title to the plural form: "4.7 Limitations of the study".

 4. In the concluding text box at the bottom of Figure 2: "...tight integration of CONCEPTUAL TRAINING and APPLIED RESEARCH RESEARCH, offering..." The word "RESEARCH" is repeated, please delete the redundant one.

 5. Please double check the language free errors.

 6. The word “manuscript” can be changed to “paper”.

Is the case presented with sufficient detail to be useful for teaching or other practitioners?

Partly

Is the work clearly and accurately presented and does it cite the current literature?

Yes

If applicable, is the statistical analysis and its interpretation appropriate?

Not applicable

Are all the source data underlying the results available to ensure full reproducibility?

Not applicable

Are the conclusions drawn adequately supported by the results?

Yes

Is the background of the case’s history and progression described in sufficient detail?

Yes

Reviewer Expertise:

Farming System

I confirm that I have read this submission and believe that I have an appropriate level of expertise to confirm that it is of an acceptable scientific standard, however I have significant reservations, as outlined above.

# Response

**Published:** 2026-05-13

**Authors:** Snezana Maletic

**Affiliations:** University of Novi Sad Faculty of Sciences, Serbia

We sincerely thank the reviewer for the careful reading of the paper and the valuable comments and suggestions. All comments were carefully considered and addressed in the revised version, as detailed below:

1. We carefully re-checked the contents of Tables 3 and 5 and identified that, in Table 5 (UNIVIE row), part of the text had been mistakenly copied from Table 3 during manuscript preparation. This has now been corrected and replaced with the appropriate content related to workshops, summer school, and webinar activities. We also re-checked the remaining entries in both tables to ensure consistency and accuracy.
2. We carefully checked the indicated sentence; however, the term “ures” was not present in the current manuscript version. Nevertheless, the paragraph was re-checked to ensure consistency and correctness.
3. The section title has been corrected to “4.7 Limitations of the study”.
4. The duplicated word “RESEARCH” in Figure 2 has been removed.
5. The paper has been carefully proofread again and additional language corrections were implemented throughout the text.
6. The word “manuscript” has been replaced with “paper” where appropriate throughout the revised version.

# Reviewer report: Reviewer response for version 1

**DOI:** 10.21956/openreseurope.24940.r71476

**Published:** 2026-04-16

**Authors:** Mohamed Hamza EL-Saeid

**Affiliations:** 1 King Saud University, Riyadh, Saudi Arabia

More recent publications should be used in this article.TwinSubDyn clearly demonstrated in this article thatThe reciprocal nature of the partnership ensured that EU institutions also gained new comparative valuable insights and knowledge, particularly regarding the environmental conditions, soil types, and amendment materials characteristic of the Western Balkans. This reciprocal exchange created a balanced partnership and established a strong foundation for continued research collaboration. Ultimately, the success of TwinSubDyn illustrates that twinning can serve as a catalyst for mutual scientific growth, institutional evolution, and durable integration into the European Research Area.

Is the case presented with sufficient detail to be useful for teaching or other practitioners?

Yes

Is the work clearly and accurately presented and does it cite the current literature?

Yes

If applicable, is the statistical analysis and its interpretation appropriate?

I cannot comment. A qualified statistician is required.

Are all the source data underlying the results available to ensure full reproducibility?

Yes

Are the conclusions drawn adequately supported by the results?

Yes

Is the background of the case’s history and progression described in sufficient detail?

Yes

Reviewer Expertise:

Chemistry and Environmental Pollution

I confirm that I have read this submission and believe that I have an appropriate level of expertise to confirm that it is of an acceptable scientific standard.

# Response

**Published:** 2026-04-16

**Authors:** Snezana Maletic

**Affiliations:** University of Novi Sad Faculty of Sciences, Serbia

We sincerely thank the reviewer for the careful reading of our manuscript and for the very positive and encouraging comments. We are particularly pleased that the reviewer recognises the relevance of the topic and the value of the reciprocal learning and collaboration achieved through the TwinSubDyn project. 

 Regarding the references, the list predominantly includes recent literature (mainly from 2023 onwards), including outputs closely related to the project and current developments in the field. References from 2020–2021 have been retained as key EU policy documents providing the strategic framework, while a small number of earlier studies (2019) were included as well-established contributions important for framing the research context during the initial phase of the project..

# Reviewer report: Reviewer response for version 1

**DOI:** 10.21956/openreseurope.24940.r71473

**Published:** 2026-03-28

**Authors:** Zijian Li

**Affiliations:** 1 Sun Yat-Sen University, Shenzhen, China

This manuscript presents a comprehensive and timely reflection on the TwinSubDyn project, addressing an important gap in the literature on capacity building within Widening countries.

 The topic is highly relevant for the European Research Area, particularly in light of ongoing efforts to reduce disparities in research performance and integration. The paper succeeds in documenting a complex, multi-institutional collaboration and offers valuable insights into how conceptual and interpretive capacity can be strengthened through structured twinning activities.

 The narrative is rich, well-informed, and grounded in extensive project experience, which makes it both informative and credible.

 Recommendations:

 Kindly add a concise statement of objectives at the end of the introduction to clearly guide the reader through the structure and purpose of the manuscript. The current introduction is rich in context, yet the specific aims are somewhat implicit.

 The authors should explain how the meta-synthesis approach was operationalized in Section 2.4 to improve transparency.

 Also, please clarify the limitations of the study in terms of scope, data type, and generalizability.

Is the case presented with sufficient detail to be useful for teaching or other practitioners?

Yes

Is the work clearly and accurately presented and does it cite the current literature?

Yes

If applicable, is the statistical analysis and its interpretation appropriate?

Yes

Are all the source data underlying the results available to ensure full reproducibility?

Yes

Are the conclusions drawn adequately supported by the results?

Yes

Is the background of the case’s history and progression described in sufficient detail?

Yes

Reviewer Expertise:

Pesticide

I confirm that I have read this submission and believe that I have an appropriate level of expertise to confirm that it is of an acceptable scientific standard, however I have significant reservations, as outlined above.

# Response

**Published:** 2026-04-16

**Authors:** Snezana Maletic

**Affiliations:** University of Novi Sad Faculty of Sciences, Serbia

We sincerely thank the reviewer for the constructive and insightful comments, which significantly improved the clarity and overall quality of the manuscript. In response, we have (i) added a concise statement of objectives at the end of the introduction, (ii) clarified the operationalisation of the meta-synthesis approach in Section 2.4, and (iii) included a paragraph addressing the study limitations. These revisions have enhanced the structure and transparency of the manuscript.
